# MicroRNA let-7c Is Downregulated in Prostate Cancer and Suppresses Prostate Cancer Growth

**DOI:** 10.1371/journal.pone.0032832

**Published:** 2012-03-30

**Authors:** Nagalakshmi Nadiminty, Ramakumar Tummala, Wei Lou, Yezi Zhu, Xu-Bao Shi, June X. Zou, Hongwu Chen, Jin Zhang, Xinbin Chen, Jun Luo, Ralph W. deVere White, Hsing-Jien Kung, Christopher P. Evans, Allen C. Gao

**Affiliations:** 1 Department of Urology, University of California Davis, Sacramento, California, United States of America; 2 Department of Biochemistry and Molecular Medicine, University of California Davis, Sacramento, California, United States of America; 3 Cancer Center, University of California Davis, Sacramento, California, United States of America; 4 Comparative Oncology Laboratory, School of Veterinary Medicine, University of California Davis, Sacramento, California, United States of America; 5 Department of Urology, Johns Hopkins University, Baltimore, Maryland, United States of America; Roswell Park Cancer Institute, United States of America

## Abstract

**Purpose:**

Prostate cancer (PCa) is characterized by deregulated expression of several tumor suppressor or oncogenic miRNAs. The objective of this study was the identification and characterization of miR-let-7c as a potential tumor suppressor in PCa.

**Experimental Design:**

Levels of expression of miR-let-7c were examined in human PCa cell lines and tissues using qRT-PCR and *in situ* hybridization. Let-7c was overexpressed or suppressed to assess the effects on the growth of human PCa cell lines. Lentiviral-mediated re-expression of let-7c was utilized to assess the effects on human PCa xenografts.

**Results:**

We identified miR-let-7c as a potential tumor suppressor in PCa. Expression of let-7c is downregulated in castration-resistant prostate cancer (CRPC) cells. Overexpression of let-7c decreased while downregulation of let-7c increased cell proliferation, clonogenicity and anchorage-independent growth of PCa cells *in vitro*. Suppression of let-7c expression enhanced the ability of androgen-sensitive PCa cells to grow in androgen-deprived conditions *in vitro*. Reconstitution of Let-7c by lentiviral-mediated intratumoral delivery significantly reduced tumor burden in xenografts of human PCa cells. Furthermore, let-7c expression is downregulated in clinical PCa specimens compared to their matched benign tissues, while the expression of Lin28, a master regulator of let-7 miRNA processing, is upregulated in clinical PCa specimens.

**Conclusions:**

These results demonstrate that microRNA let-7c is downregulated in PCa and functions as a tumor suppressor, and is a potential therapeutic target for PCa.

## Introduction

Prostate cancer (PCa) is the most commonly occurring malignancy and the second most frequent cause of cancer-related mortality in men in the US. The majority of patients with advanced PCa respond initially to androgen deprivation therapy, but relapse due to the growth of castration resistant prostate cancer (CRPC) cells. Significant efforts have been focused on understanding the mechanisms involved in the development and progression of CRPC. MicroRNAs (miRNAs) are endogenous small non-coding RNAs that can interfere with protein expression either by inducing cleavage of their specific target mRNAs or by inhibiting their translation. Mature miRNAs are evolutionarily conserved ∼22nt single stranded RNAs resulting from a multistep processing of larger precursor molecules via Drosha and Dicer. Eventually the mature miRNAs are incorporated into the RISC complex along with their target mRNAs which are recognized by sequence complementarity in the 3’-UTR [1]. Each miRNA can target several different mRNAs and each mRNA can be targeted by multiple miRNAs, generating an intricate network of gene expression regulation. MiRNAs have been shown to regulate a rapidly increasing list of complex biological processes including differentiation, cell cycle, apoptosis and metabolism [2]. An overwhelming amount of new evidence points to their roles as tumor suppressors or oncogenes which presents a whole spectrum of novel diagnostic and therapeutic opportunities [1], [3].

Let-7 encodes an evolutionarily conserved family of 13 homologous miRNAs located in genomic locations frequently deleted in human cancers [4]. Expression of let-7 in lung cancer cell lines reduced cell proliferation [5] and inhibited tumorigenesis of breast cancer cells while also reducing metastases [6]. Overexpression of let-7 also decreased lung cancer cell resistance to radiation therapy [7]. Reduced expression of let-7 in human lung cancers has been associated with shortened post-operative survival, suggesting that let-7 may be an important prognostic marker in lung cancer [8]. Similarly, 5-year progression free survival rate was found to be higher in ovarian cancer patients with lower HMGA2/let-7 ratios compared to those with higher HMGA2/let-7 ratios [9]. There is an evident link between loss of let-7 expression and development of poorly differentiated and aggressive cancers [10]. Let-7 expression was found to be downregulated in localized PCa tissues relative to benign peripheral zone tissue [11], [12]. Let-7 members have been shown to regulate expression levels of oncogenes like HMGA2 [5], RAS [13] and Myc [14] along with genes involved in cell cycle and cell division regulation. Therapeutic strategies are being developed targeting let-7, using either lenti-or adeno-viral-encoded overexpression of let-7 or transient transfection of double-stranded precursors of let-7 [15], [16]. Thus, let-7 shows promise as a molecular marker in certain cancers and as a potential therapeutic in cancer treatment.

Lin28, a highly conserved RNA-binding protein and a master regulator of let-7 miRNA processing, is overexpressed in primary human tumors [17], [18] and is postulated to be one of the embryonic stem cell factors that promote oncogenesis and proliferation of cancer cells, by repression of the let-7 family of tumor suppressors [19]. Lin28 binds to the terminal loops of the precursors of let-7 family miRNAs and blocks their processing into mature miRNAs [20], [21]. Lin28 also derepresses c-Myc by repressing let-7 and c-Myc transcriptionally activates Lin28 [22], [23]. This Lin28/let-7/c-Myc loop may play an important role in the deregulated miRNA expression signature observed in many cancers [24].

In this study, we demonstrate that let-7c, one of the members of the let-7 family, suppresses PCa growth *in vitro* and *in vivo*. Overexpression of let-7c led to inhibition of anchorage-dependent as well as anchorage-independent growth of PCa cells. Reexpression of let-7c in xenografts of human PCa cells using lentivirally encoded let-7c inhibited tumor growth significantly. In addition, let-7c expression is downregulated in clinical PCa specimens. Our results imply that prostate tumor growth is regulated by let-7c and that reconstitution of let-7 may have beneficial effects in PCa by decreasing survival and proliferation of tumor cells.

## Materials and Methods

### Cell lines and Reagents

LNCaP, C4-2B, PC-346C and DU145 cells were obtained from the American Type Culture Collection (ATCC, Manassas, VA). LNCaP, C4-2B and PC346C cells are androgen receptor (AR) positive and respond to androgen supplementation, while DU145 cells are AR negative and are androgen-insensitive. LNCaP-S17 and LNCaP-IL6+ cell lines were generated in our laboratory by stable transfection of full length IL-6 cDNA into LNCaP cells and by chronic treatment of LNCaP cells with 5 ng/ml recombinant IL-6 respectively. Both cell lines exhibit autocrine IL-6 signaling. Antibodies against Actin and Tubulin were purchased from Santa Cruz Biotechnologies (Santa Cruz, CA). Lin28 antibodies were obtained from Abcam (San Francisco, CA). SYBR Green iQ Supermix was from Bio-Rad. Lentivector-based let-7c construct was obtained from System Biosciences and Lin28 ORF and shRNA constructs were obtained from Open Biosystems.

### Western Blot Analysis

Cells were lysed in high salt buffer containing 50 mM Hepes pH 7.9, 250 mM NaCl, 1 mM EDTA, 1% NP-40, 1 mM PMSF, 1 mM Na Vanadate, 1 mM NaF and protease inhibitor cocktail (Roche) as described earlier [25]. Total protein was estimated using the Coomassie Protein Assay Reagent (Pierce, Rockford, IL). Equal amounts of protein were loaded on 10% SDS–PAGE and transferred to nitrocellulose membranes. The membranes were blocked with 5% nonfat milk in PBST (1x PBS+0.1% Tween-20) and probed with primary antibodies in 1% BSA. The signal was detected by ECL (GE Healthcare) after incubation with the appropriate HRP-conjugated secondary antibodies.

### Measurement of PSA

PSA levels were measured in the culture supernatants using ELISA (United Biotech Inc, Mountain View, CA) according to the manufacturer’s instructions.

### Real-Time Quantitative RT-PCR

LNCaP cells were transfected with the indicated plasmids or oligonucleotides and total RNAs were extracted using Trizol reagent (Invitrogen). cDNAs were prepared after digestion with RNase-free RQ1 DNase (Promega). The cDNAs were subjected to real-time reverse transcription-PCR (RT-PCR) using SYBR Green iQ Supermix (Bio-Rad) according to the manufacturer’s instructions and as described previously [26], [27]. Reactions were performed with 1 µL RT-PCR cDNA, 0.5 µL each of forward and reverse primers (10 µmol/L), 10.5 µL double-distilled water, and 12.5 µL iQ SYBR Green supermix. Each reaction was normalized by coamplification of actin. Triplicates of samples were run on default settings of Bio-Rad CFX-96 real-time cycler. Primers used for quantification of Lin28 were: Forward 5′- GCCCCTTGGATATTCCAGTC-3′ and Reverse 5′-AATGTGAATTCCACTGGTTCTCCT-3′. miRNA qPCR was performed with the use of miRCURY LNA Universal RT microRNA PCR kit and LNA-conjugated miRNA primers (Exiqon) according to manufacturer’s instructions.

### Northern Blotting

20 µg each of total RNAs from LNCaP, PC-3, DU145, LNCaP-S17 and LNCaP-IL6+ cells were run on 15% Urea-PAGE gels and transferred to nylon membranes. After UV cross linking, membranes were hybridized to a probe recognizing the mature sequence of let-7c. U6 snRNA was used as loading control.

### miRNA in Situ Hybridization

*In situ* hybridization (ISH) was performed to determine the patterns of expression of let-7c in human clinical PCa tissue microarray containing 160 cores each from unmatched benign and cancerous prostates. ISH was performed using the locked nucleic acid (LNA)-conjugated let-7c-specific probe from Exiqon according to manufacturer’s instructions.

### Clonogenic Assays

Anchorage-dependent clonogenic ability assays were performed as described previously [28]. Briefly, LNCaP cells transfected with either empty vector or let-7c were seeded at low densities (400 cells/dish) in 10 cm culture plates. The plates were incubated at 37^o^C in media containing either 10% FBS or 10% charcoal-stripped FBS (CS-FBS) and were left undisturbed for 14 days. At the end of the experiment, cells were fixed with methanol, stained with crystal violet and the numbers of colonies were counted.

### Soft-agar Colony Formation Assays

Anchorage-independent colony formation assays were performed using C4-2B and LNCaP-S17 cells transfected with the indicated plasmids. After transfection, cells were plated in 0.35% agarose overlying a 1.2% agar layer. Cells were fed twice a week with complete RPMI1640 and were incubated at 37^0^C for 2 weeks. At the end of the experiment, colonies were stained with 0.005% Crystal Violet and counted.

### Cell Growth Assays

LNCaP, C4-2B, DU145, LNCaP-S17 and LN-IL6+ cells were transfected with plasmids expressing let-7c and viable cell numbers were determined at 0, 24 and 48 h using a Coulter cell counter.

**Figure 1:**
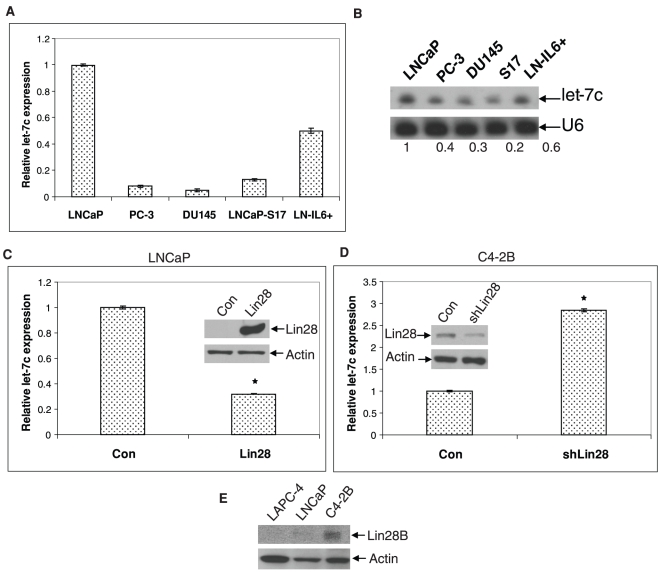
Let-7c is expressed in PCa cells. **A**) Total RNAs from LNCaP, PC-3, DU145, LNCaP-S17 and LN-IL6+ cells were analyzed by qRT-PCR. Results are presented as relative fold change compared to expression levels in LNCaP. Data points represent mean ± SD of triplicate samples from two independent experiments. **B**) 20 µg each of the above RNAs were also analyzed by northern blotting. U6 snRNA was used as the loading control. **C**) qRT-PCR showing the decrease in let-7c expression in LNCaP cells expressing Lin28 compared to LNCaP cells transfected with the empty vector (Con). Inset Western blot shows expression of Lin28. **D**) qRT-PCR showing the increase in let-7c expression in C4-2B cells transfected with shRNA against Lin28 compared to C4-2B cells transfected with control GFP shRNA. Inset Western blot shows downregulation of Lin28. Data points represent mean ± SD of triplicate samples from two independent experiments. Error bars denote ± SD (*p < 0.05). **E**) Western blot showing the expression levels of Lin28 in PCa cells. Actin is used as a loading control.

### Apoptosis Assays using Cell Death Detection ELISA

DNA fragmentation in LNCaP, DU145, LNCaP-S17 and LN-IL6+ cells transfected with plasmids as indicated in figures was assessed by the Cell death detection ELISA kit (Roche, Indianapolis, IN) according to the manufacturer’s instructions.

### Generation of Stable Cell Lines

Stable cell lines of LNCaP and C4-2B expressing let-7c were generated by transfection of plasmids containing the cDNAs and selection of clones after application of selective pressure with appropriate antibiotics.

**Figure 2:**
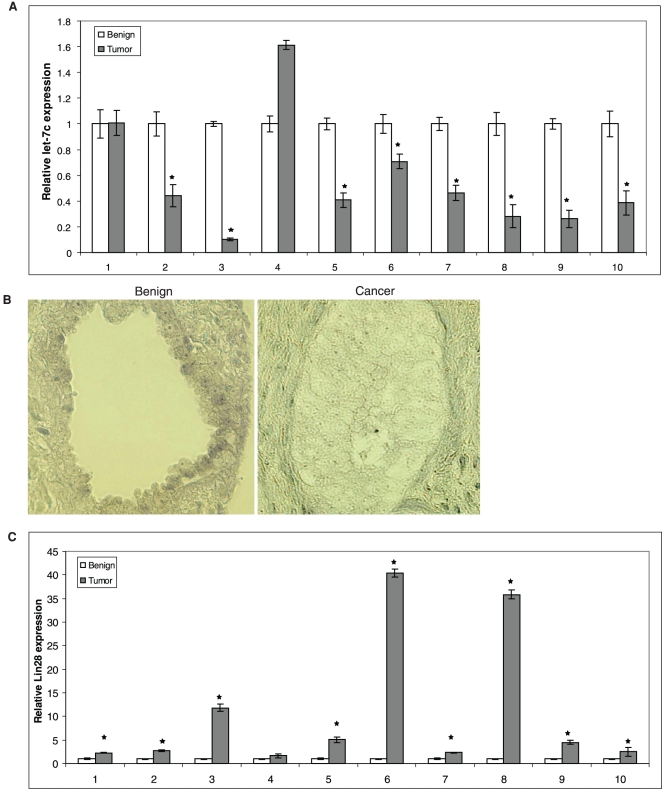
Let-7c expression is downregulated in human PCa. **A**) Relative expression levels of let-7c were measured by qRT-PCR in total RNAs extracted from 10 paired benign and tumor human prostate samples. **B**) Let-7c levels were measured using *in situ* hybridization in TMAs containing 160 cores each from unmatched benign and cancerous prostate biopsies. Representative images are shown for benign and cancer cores. Expression of let-7c was higher in benign prostates compared to cancerous prostates. **C**) Relative expression levels of Lin28 in the 10 paired benign and tumor human prostate samples. Expression levels of Lin28 were correlated inversely with those of let-7c. Error bars denote ± SD (*p < 0.05).

**Figure 3:**
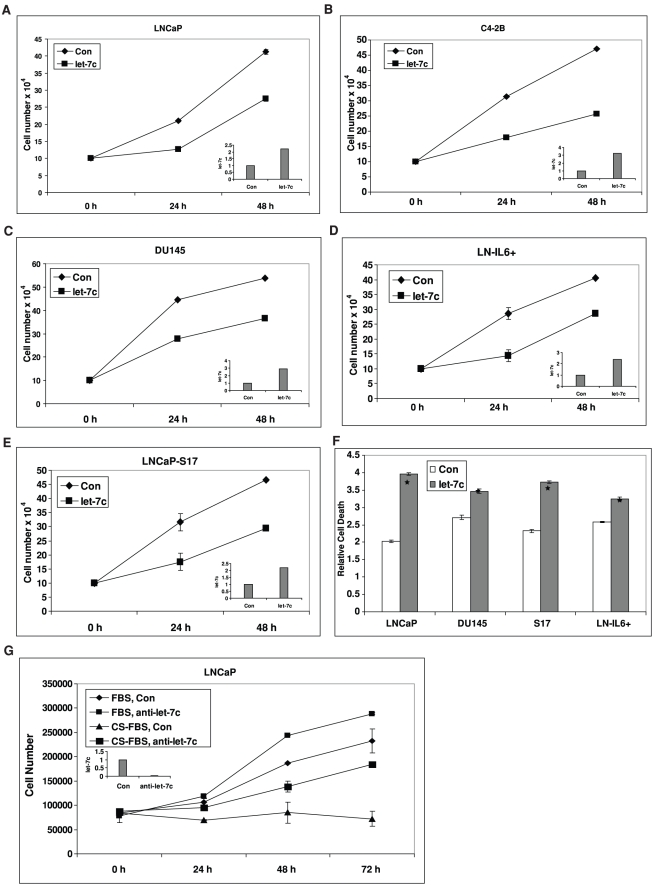
Let-7c inhibits growth of human PCa cells *in vitro*. LNCaP (**A**), C4-2B (**B**), DU145 (**C**), LN-IL6+ (**D**) and LNCaP-S17 (**E**) cells were transfected with let-7c or empty vector (Con) and cell numbers were determined after 24 and 48 h. Growth of PCa cells was suppressed by let-7c. Insets show the levels of expression of let-7c plasmid. **F**) Cell death was analyzed in LNCaP, DU145, LNCaP-S17 and LN-IL6+ cells transfected with let-7c or empty vector (Con). Let-7c induced apoptotic cell death of prostate cancer cells. **G**) LNCaP cells transfected with anti-sense oligos against let-7c or scrambled oligos (Con) were grown in FBS and CS-FBS and cell numbers determined. Inset shows the downregulation of expression of let-7c by anti-sense oligos. LNCaP cells with downregulated expression of let-7c exhibited faster growth in CS-FBS compared to controls. Data points represent mean ± SD of triplicate samples from two independent experiments. Error bars denote ± SD (*p < 0.05).

### Animals

6–8 week old male nude mice were maintained in the Animal Facility at the UC Davis Medical Center. All experimental procedures using animals were approved by the Institutional Animal Care and Use Committee of UC Davis. 1–2×10^6^ cells/flank were injected s.c. into both flanks and tumors were allowed to grow. Once the tumors reached 0.5 cm^3^, 1x10^7^ ifu (infectious units) of lentiviruses containing either empty vector with GFP or let-7c precursor were injected intratumorally. At the end of the experiments, mice were sacrificed and tumors were excised. Sera were collected for measurement of PSA.

### Human PCa Specimens

Paired benign and tumor prostate tissues were prepared as described previously [29]. Surgical specimens used in this study were radical prostatectomy specimens (one from robotic surgery) collected at the Johns Hopkins University from 2002 to 2007. Specimens were selected from the frozen prostate tumor bank if paired frozen blocks enriched for histologically normal and tumor areas were available. Frozen blocks were manually trimmed to further enrich the histology of interest. Cryosections (7 µm) were prepared from each block before RNA extraction. The tumor content in the tumor specimens was greater than 80%, whereas normal samples had at least 60% epithelium content and no evidence of tumor present. The first and last sections for each block were H&E stained and used for % epithelium calculation. The use of de-identified surgical specimens for molecular analysis was approved by the IRB.

### Statistical Analyses

Data are shown as means ± SD. Multiple group comparison was performed by one-way ANOVA followed by the Scheffe procedure for comparison of means. *P*<0.05 was considered significant.

## Results

### Let-7c is Expressed in PCa Cells

Our previous studies using miRNA microarrays showed that let-7c was among the most commonly modulated miRNAs in PCa cells (unpublished data). To determine the relative levels of expression of let-7c in PCa cells, we isolated total RNAs from LNCaP (androgen-dependent, AR-positive), PC-3, DU145 (castration-resistant, AR-negative) cells as well as LNCaP-S17 cells expressing IL-6 [30] and LN-IL6+ cells chronically treated with IL-6 [31]. cDNAs were analyzed by quantitative RT-PCR using primers amplifying the mature form of let-7c (Exiqon) specifically. Our results showed that let-7c is expressed at high levels in LNCaP cells compared to the hormone-refractory PC-3 and DU145 cells (Fig. 1A). Earlier reports showed that IL-6 and let-7 exhibit reciprocal regulation of expression. LNCaP-IL6+ and LNCaP-S17 cells (autocrine IL-6 signaling) showed reduction in let-7c levels, consistent with the report that IL-6 reduces let-7 expression in PCa cells and that let-7 regulates IL-6 expression [18]. These results were also confirmed by northern blotting using a probe against the mature let-7c sequence (Fig. 1B), suggesting that let-7c levels are reduced in more aggressive and castration-resistant PCa cells.

Recent reports showed that expression of let-7 family of miRNAs is regulated by Lin28, a master regulator of miRNA processing [18], [20]. Consistent with the finding, we found that overexpression of Lin28 led to a reduction in let-7c levels in LNCaP cells (Fig. 1C), while downregulation of Lin28 using Lin28 shRNA led to an increase in let-7c levels (Fig. 1D) in C4-2B cells which express endogenous Lin28 (Fig. 1E). Downregulation or overexpression of Lin28 was confirmed by Western blotting (Inset Fig. 1C&D). These results show that Lin28 plays a critical role in regulation of let-7c expression in PCa cells.

### Let-7c Expression is Downregulated in Human PCa

To determine whether the levels of let-7c expression are downregulated in clinical PCa, we analyzed RNAs from 10 paired benign and tumor human PCa specimens by quantitative RT-PCR. RNAs were isolated from human tissues, reverse transcribed and subjected to qRT-PCR using LNA-conjugated let-7c primers (Exiqon). The levels of let-7c were significantly decreased in 8/10 tumors compared to their matched benign prostate tissues (Fig. 2A). We also analyzed two tissue microarrays containing benign and cancerous prostate biopsies respectively by *in situ* hybridization using LNA-conjugated mature let-7c-specific probe (Exiqon). The images were analyzed using an Olympus IX81 microscope and DP Controller Software. Our results showed that let-7c was highly expressed in benign PCa, while its expression was downregulated in the cancerous prostate (Fig. 2B). Collectively, these results suggest that loss of let-7c expression may be associated with prostate tumorigenesis.

Since Lin28 is a key regulator of let-7c expression, we examined Lin28 expression in the 10 paired benign and tumor prostate samples by qRT-PCR using primers which amplify Lin28 mRNA specifically. Expression levels of Lin28 were found to be significantly elevated in 9/10 pairs of matched benign and tumor prostate specimens (Fig. 2C). Expression of Lin28 was correlated inversely with expression of let-7c with a correlation coefficient of -0.4, suggesting that let-7c expression is regulated primarily by Lin28 in human PCa.

### Let-7c Decreases the Growth of PCa Cells in Vitro

To determine whether let-7c affects the growth of PCa cells, LNCaP, C4-2B, DU145, LNCaP-S17 and LN-IL6+ cells were transfected with plasmids encoding let-7c or empty vector and cell numbers were counted after 24 and 48 h. Cell numbers of all PCa cell lines overexpressing let-7c were reduced by ∼40% at 48 h (Fig. 3A-E). Insets show the levels of expression of let-7c plasmid in these cells. To determine whether the observed decrease in cell growth was due to apoptotic cell death, DNA fragmentation was analyzed by Cell Death Detection ELISA. As shown in Fig. 3F, apoptosis in cells overexpressing let-7c was enhanced compared to the controls, suggesting that the inhibition in cell growth induced by let-7c is partly due to increased apoptotic cell death.

**Figure 4:**
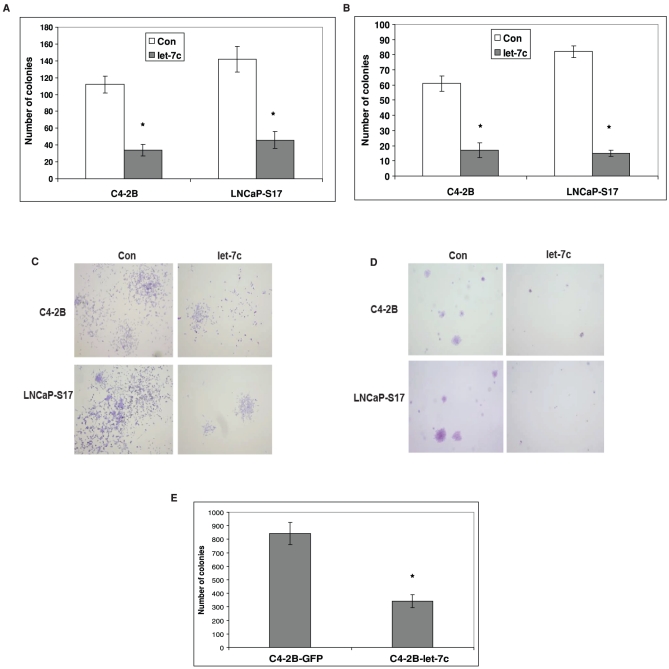
Let-7c inhibits colony forming abilities of human PCa cells. Clonogenic (**A**) and soft agar colony forming (**B**) abilities of LNCaP-S17 and C4-2B cells transfected with let-7c or empty vector (Con) were assayed. Let-7c inhibited cell growth and survival in anchorage-dependent and –independent conditions. **C**) Clonogenic assay-Upper and lower panels represent colony sizes of C4-2B and LNCaP-S17 cells expressing control (empty vector) or let-7c respectively. **D**) Soft agar assay-Upper and lower panels represent colony sizes of C4-2B and LNCaP-S17 cells expressing control (empty vector) or let-7c respectively. **E**) Number of colonies formed in clonogenic assay by C4-2B cells stably expressing let-7c. Let-7c decreased the number of colonies formed by the PCa cells. Data points represent mean ± SD of triplicate samples from two independent experiments. Error bars denote ± SD (*p < 0.05).

In addition, we tested whether downregulation of let-7c would enhance the ability of androgen-sensitive PCa cells to grow in androgen-deprived conditions. We transfected anti-sense oligos against let-7c or control scrambled oligos into LNCaP cells supplemented with either FBS or charcoal-stripped FBS (CS-FBS) and monitored cell growth. The results demonstrated that downregulation of let-7c by anti-sense promoted androgen-dependent LNCaP cell growth in conditions of androgen deprivation (Fig. 3G). Downregulation of let-7c by the anti-sense oligos was confirmed using qRT-PCR (Fig. 3G, inset). These findings suggested that castration-resistant growth of PCa may be characterized by downregulation of let-7c expression.

**Figure 5:**
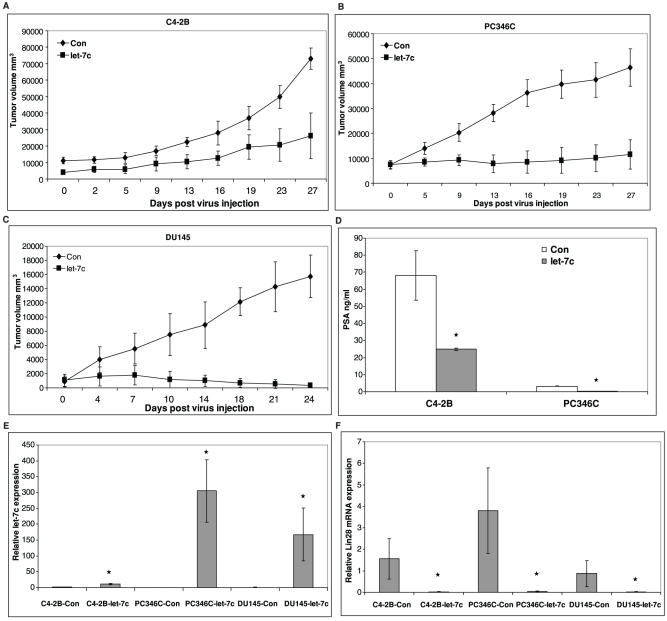
Let-7c suppresses tumor growth of human PCa xenografts *in vivo*. C4-2B (**A**), PC346C (**B**) and DU145 (**C**) cells were injected into both flanks of nude mice and the tumors received a single intratumoral injection of lentiviruses expressing either GFP (control) or let-7c. Tumor growth was monitored twice weekly over 3 weeks. Data points represent mean ± SD of tumor volume (mm^3^) of all mice at the indicated time points. **D**) Secretion of PSA by C4-2B and PC346C xenografts was measured in mouse sera by ELISA. Reconstitution of let-7c in the tumors reduced secretion of PSA by the xenografts. At the end of the experiments, tumor tissues were excised, total RNAs prepared and subjected to qRT-PCR to assess mRNA levels of let-7c (**E**) and Lin28 (**F**). Error bars denote ± SD (*p < 0.05).

We also analyzed clonogenic ability of PCa cells expressing let-7c in both anchorage-dependent and anchorage-independent conditions. LNCaP-S17 and C4-2B cells were transfected with let-7c or empty vector and colony formation assays were performed. Both clonogenic (Fig. 4A) and soft agar colony (Fig. 4B) formation abilities of LNCaP-S17 and C4-2B cells were suppressed by overexpression of let-7c. The number of colonies formed on substrata by LNCaP-S17 cells was reduced from 142±15 to 46±10 and the number of colonies formed by C4-2B cells was reduced from 122±10 to 34±7 (Fig. 4A). Similarly, the number of colonies formed in soft agar by LNCaP-S17 cells was reduced from 82±4 to 15±2 and the number of colonies formed by C4-2B cells was reduced from 61±5 to 21±5 (Fig. 4B) by overexpression of let-7c. Furthermore, the size of colonies formed by control-transfected cells was larger compared to the colonies formed by let-7c-transfected cells (Fig. 4C&D). These results suggest that let-7c may inhibit PCa cell growth in anchorage-dependent as well as –independent conditions. These findings were also confirmed with clonogenic assay in C4-2B cells stably expressing let-7c (Fig. 4E).

### Let-7c Inhibits Tumor Growth of Human PCa Cell Xenografts

We generated lentiviruses encoding GFP-tagged let-7c precursor using the Lentivector Expression System (System Biosciences). To determine whether let-7c exhibits anti-proliferative effects on PCa xenografts *in vivo*, we injected 2x10^6^ C4-2B or PC346C (both cell lines are AR-positive) cells s.c. into both flanks of male nude mice and monitored tumor development. Once tumors reached the size of 0.5 cm^3^, mice were randomized into two groups. The experimental mice received a single intratumoral injection of lentivirally encoded let-7c, while control mice received lentiviruses expressing GFP. Tumor growth was monitored over 3 weeks, with tumor measurements twice weekly. At the end of 3 weeks, tumors were excised, RNAs prepared and qRT-PCR performed to assess levels of let-7c in the xenografts. The results showed that tumor growth of C4-2B (Fig. 5A) and PC346C (Fig. 5B) xenografts was inhibited significantly in mice injected with let-7c-containing lentiviruses compared to mice injected with control lentiviruses. In addition, we also tested whether let-7c can suppress tumor growth of AR-negative xenografts. We injected 1x10^6^ DU145 cells/flank s.c. into male nude mice and performed similar experiments with half the mice receiving a single intratumoral injection of lentiviruses encoding let-7c and the other half receiving lentiviruses encoding the empty vector. The results showed that let-7c was successful in suppressing tumor growth of DU145 xenografts similar to C4-2B or PC346C xenografts (Fig. 5C). Levels of PSA, a classic target gene of AR, secreted by the AR-positive xenografts were measured in the mouse sera using a human-specific PSA ELISA kit and were normalized to tumor weights. Results showed that injection of let-7c-expressing lentiviruses reduced the secretion of PSA by the tumor xenografts of C4-2B and PC346C compared to control lentiviruses (Fig. 5D). qRT-PCR showed that let-7c levels were enhanced in the tumors injected with let-7c-encoding lentiviruses, while levels of Lin28 were reduced (Fig. 5E&F). These findings suggest that overexpression of let-7c suppresses prostate tumor growth, and that reconstitution of let-7c levels may present an attractive therapeutic strategy against human PCa.

## Discussion

In this study, we found that let-7c expression is downregulated in castration-resistant PCa cells and clinical specimens. Transfection of lentiviral-encoded let-7c inhibited the growth and clonogenicity of PCa cells while enhancing apoptotic cell death. Conversely, downregulation of let-7c by anti-sense oligonucleotides conferred a survival advantage on PCa cells in androgen-replete as well as androgen-depleted environments. Intratumoral injection of lentivirally encoded let-7c inhibited PCa xenograft tumor growth, demonstrating that let-7c functions as a tumor suppressor that inhibits prostate tumor growth. These results suggest that miRNA let-7c plays an important role in PCa cell proliferation and may be exploited for therapeutic applications.

The mechanisms of suppression of prostate tumor growth by let-7c may include direct or indirect regulation of expression levels of oncogenes such as Myc and Lin28. In a recent report, we showed that let-7c targets the expression of the AR via targeting the expression of Myc [32]. The AR is a key survival factor for prostate tumor cells and reduction of its expression has been demonstrated to suppress prostate tumor growth [33]. In confirmation of these findings, expression of PSA, one of the classic target genes of the AR, was found to be suppressed by let-7c in xenografts of C4-2B and PC346C cells in this study.

It is well documented that Lin28 plays a major role in regulation of let-7c expression [18], [21]. This is consistent with our studies showing that Lin28 suppresses let-7c expression in PCa cells. Overexpression of Lin28 inhibited let-7c expression in LNCaP cells, while knockdown of Lin28 expression increased let-7c in C4-2B cells. In addition, we demonstrated that overexpression of let-7c reduced the levels of Lin28 expression in the tumors of PCa xenograft models (Fig. 5F), indicating that a negative feedback loop exists between Lin28 and let-7c.

Several reports have established the important role of let-7, showing that members of the let-7 family are downregulated in lung cancers and that this downregulation is correlated with poor survival [8]. A tumor suppressor role has been attributed to the let-7 family of miRNAs and appears to be undisputed except in rare cases, such as let-7a, which has been reported to target caspase-3 in human cancers [34], thus suppressing susceptibility of cancer cells to chemotherapeutic-induced cell death. In malignant mesothelioma, let-7b* was found to be highly expressed [35] and the upregulation of let-7b and let-7i was associated with high grade transformation in lymphoma [36]. These conflicting data on deregulation of let-7 in various human cancers show that individual let-7 family members may have distinct and varying activities in different cells and do not simply exhibit redundant functions.

An earlier study by Dong et al. [37] reported that let-7a suppresses prostate tumor growth by targeting E2F2 and CCND2. Our studies suggest that let-7c suppresses prostate tumor growth by several pathways including regulation of IL-6, Myc, Lin28 and the AR [32]. In addition, direct reconstitution of let-7c by injection of lentivirally encoded let-7c into established xenograft tumors marks an important step in the direction of prospective therapeutic strategies using let-7c. Even though members of the let-7 family may exhibit some redundant functions, individual components may be subject to differential and tissue-specific regulation in different cell types. Our results with lentivirally encoded let-7c show that, if feasible strategies to reconstitute let-7c in prostate tumors can be developed, reconstitution of let-7c may represent a potential therapeutic agent for PCa treatment.

In summary, our study demonstrates that the miRNA let-7c plays an important role in inhibition of PCa cell proliferation and castration-resistant progression. Downregulation of expression of Let-7c and the let-7c/Lin28 feedback loop may facilitate prostate tumor growth. Targeting this novel pathway may pave the way for effective therapeutic strategies in PCa therapy.
